# Novel Cytochrome P450-3A4 Enzymatic Nanobiosensor for Lapatinib (a Breast Cancer Drug) Developed on a Poly(anilino-co-4-aminobenzoic Acid-Green-Synthesised Indium Nanoparticle) Platform

**DOI:** 10.3390/bios13090897

**Published:** 2023-09-21

**Authors:** Jaymi Leigh January, Ziyanda Zamaswazi Tshobeni, Nokwanda Precious Pearl Ngema, Abongile Nwabisa Jijana, Emmanuel Iheanyichukwu Iwuoha, Takalani Mulaudzi, Samantha Fiona Douman, Rachel Fanelwa Ajayi

**Affiliations:** 1 SensorLab (UWC Sensor Laboratories), University of the Western Cape, Private Bag X17, Bellville, Cape Town 7535, South Africa; 2 Nanotechnology Innovation Centre, Advanced Materials Division, Mintek, Private Bag X3015, Randburg, Johannesburg 2125, South Africa; 3 Department of Biotechnology, University of the Western Cape, Private Bag X17, Bellville, Cape Town 7535, South Africa; 4 Department of Chemistry, University of Cape Town, Private Bag X3, Rondebosch, Cape Town 7701, South Africa

**Keywords:** biosensor, breast cancer drug (lapatinib), cytochrome P450-3A4, green-synthesized indium nanoparticles, polyaniline

## Abstract

Breast cancer (BC) is one of the most common types of cancer disease worldwide and it accounts for thousands of deaths annually. Lapatinib is among the preferred drugs for the treatment of breast cancer. Possible drug toxicity effects of lapatinib can be controlled by real-time determination of the appropriate dose for a patient at the point of care. In this study, a novel highly sensitive polymeric nanobiosensor for lapatinib is presented. A composite of poly(anilino-co-4-aminobenzoic acid) co-polymer {poly(ANI-co-4-ABA)} and coffee extract-based green-synthesized indium nanoparticles (InNPs) was used to develop the sensor platform on a screen-printed carbon electrode (SPCE), i.e., SPCE||poly(ANI-co-4-ABA-InNPs). Cytochrome P450-3A4 (CYP3A4) enzyme and polyethylene glycol (PEG) were incorporated on the modified platform to produce the SPCE||poly(ANI-co-4-ABA-InNPs)|CYP3A4|PEG lapatinib nanobiosensor. Experiments for the determination of the electrochemical response characteristics of the nanobiosensor were performed with cyclic voltammetry (CV) and differential pulse voltammetry (DPV). The nanobiosensor calibration for 0–100 ng/mL lapatinib was linear and gave limit of detection (LOD) values of 13.21 ng/mL lapatinib and 18.6 ng/mL lapatinib in physiological buffer and human serum, respectively. The LOD values are much lower than the peak plasma concentration (C_max_) of lapatinib (2.43 µg/mL), which is attained 4 h after the administration of a daily dose of 1250 mg lapatinib. The electrochemical nanobiosensor also exhibited excellent anti-interference performance and stability.

## 1. Introduction

Globally, cancer is regarded as the leading cause of death and morbidity, wherein it can also be considered as an epidemic [1]. Amongst the many types of cancers, breast cancer (BC) is considered to be one of the most distressing diseases to date. Following treatment with standard chemotherapy, many women with advanced or metastatic BC can only expect to have limited therapeutic options. In this advanced setting, there has been a need for alternative treatments, such as laser therapy [2], photodynamic therapy [3], targeted therapy [4], hormone therapy [5], and immunotherapy [6]. Amongst these, targeted therapy enables cancer cells to be marked, allowing them to be easily found and destroyed by the immune system [7]. This type of therapy has been reported to have a 50% higher effectiveness than chemotherapy, highlighting the significance of studying targeted therapy drugs or substances. Lapatinib (LAPA) is a type of targeted therapy that is used for treating various types of cancer [8], where it has been shown to be effective in treating patients diagnosed with BC. It is known that through the detection of a drug and monitoring of a drug’s concentration in a patient’s body, it will help in the development of a personalized treatment plan [9]. This plan will then enable patients to receive the most appropriate dosage, resulting in a lower possibility of side effects. However, the widely used sensitive and precise monitoring/detection methods, such as sequencing [10], polymerase chain reaction, and flow cytometry [11], are strenuous, complex, and expensive procedures [12]. To satisfy the demand, analysis techniques that are selective, rapid, and sensitive have gained considerable attention over the past few years. Recent studies have shown that the use of electrochemical detection techniques for controlling a disease is an essential step in addressing our current issues. Amongst these techniques, biosensors offer multiplexed measurements, high sensitivity and selectivity, reduced time for sample preparation, and can be used for point-of-care analysis, and will, therefore, play a huge role in cancer management [13]. Electrochemical biosensors are analytical devices that can convert biological information into an electrical signal. The principle of this device is based on the interaction between the analyte and the biological recognition element as it will then produce a sensor signal because of the electrical properties that are being changed due to the redox reaction taking place during this interaction [14]. Despite the many advantages of these biosensors, they still have some drawbacks. Thus, extensive research is still being done on the improvement of the overall performance of biosensors, through material studies, synthetic routes, and immobilization chemistry.

The use of nanoparticles (NPs) in biosensors to identify cancer carries the promise of improving the survival rate of patients. Due to their various properties, nanoparticles have been widely used in sensing systems to detect and monitor different diseases and viruses [15,16,17,18,19,20,21]. Likewise, there are a variety of approaches that can be used to create these nanoparticles. Amongst them, the use of a green-synthesis approach has received widespread acceptance for its energy efficiency, low cost, and minimal environmental contamination from byproducts [22]. This method does not require any stabilizers and makes use of various organic non-toxic materials. For instance, the key phenolic compounds found in plant extracts have been used as bio-reductants for the formation of NPs [23]. Green-synthesized nanomaterials have many areas of applications, such as in textiles [24], drug delivery systems [25], cosmetics [26], and diagnostic medicine [27], making NPs to be among the most versatile sustainable materials to date.

The integration of these green-synthesized nanoparticles and conducting polymers (CP) can be an excellent complement of one another as they both possess many benefits, such as improved electrical conductivity [28]. Polyaniline (PANI) is a promising CP that has attracted attention due to its low cost and stability [29]. It can be synthesized through various methods, such as electrochemical or chemical processes. Even though PANI has good properties, its applicability in biochemical systems is still restricted. Researchers have shown that these issues can be solved by modifying the polyaniline chain with various functional groups. These groups can then be used to co-polymerize meta, ortho, or para-basic (amino) aniline polymers. Thus, the combination of nanoparticles and conducting co-polymeric films will enhance the electronic properties of these films which will make them useful for various sensor applications [30,31,32,33].

To date, there are no known electrochemical methods for detecting LAPA on screen-printed carbon electrodes (SPCE), however, there have been studies that utilize both electroanalytical and electrochemical methods for quantifying LAPA on conventional glassy carbon electrode (GCE). Topal et al. [34] conducted a study on the electro-oxidation of LAPA and the interaction between it and double stranded DNA on a modified GCE, while Aksoz et al. [35] used their proposed modified GCE and found that the substance could be detected with a limit of 5.71 nM. A similar study conducted by Mathad et al. [36] utilized a β-cyclodextrin-based modified GCE for the LAPA sensing process and obtained the lowest limit of detection of 0.181 nM. Additionally, there has been only one electroanalytical procedure [37] for detecting LAPA on a GCE. Thus, in comparison, we have fabricated an electrochemical sensing platform that is sensitive and simple for detecting LAPA at nanomolar levels using a co-polymer nanocomposite on SPCE.

Herein, we present the development and calibration of a novel polymeric nanobiosensor for lapatinib, prepared with poly(anilino-co-4-aminobenzoic acid)|indium nanoparticles (poly(ANI-co-4-ABA-InNPs)) and cytochrome p450-3A4 (CYP3A4) on SPCE. The analytical performance of the nanobiosensor was studied by differential pulse voltammetry (DPV) in both physiological buffer and real sample solutions.

## 2. Materials and Methods

### 2.1. Materials

Merck (Pty) Ltd, Johannesburg, South Africa was the source for all the reagents (except the coffee beans), which were received and used as analytical grade chemicals. The reagents include human serum (human male AB plasma), CypExpress™ 3A4 Cytochrome P450 human (Merck product no. MTOXCE3A4-250MG; the CYP3A4 was expressed in *Picha pastoris*) stored in the freezer at −80 °C when not in use, indium (III) nitrate hydrate, sulfuric acid (H_2_SO_4_, 99.99%), glutaraldehyde (GA; 25%—diluted to a 1.25% stock aqueous solution), aniline (pure), 4-aminobenzoic acid, 6-mercapto-1-hexanol (MCH), polyethylene glycol (PEG), bovine serum albumin (BSA), 10 mM Dulbecco’s phosphate-buffered saline (DPBS; pH 7.4), dimethyl sulfoxide (DMSO; ≥99.5%), and dimethylformamide (DMF; ≥99.9%). Lapatinib powder (C_29_H_26_ClFN_4_O_4_S; ≥98% (HPLC)) was used to prepare 1 mg/mL stock solution in a 1:1 ratio by volume mixture of DMSO and DMF. Thereafter, appropriate dilutions of LAPA were prepared with 0.1 M PBS solution (pH 7.4). 100% Arabica coffee beans (single origin Kilimanjaro) were purchased from Woolworths, Cape Town, South Africa. 0.1 M phosphate-buffered saline (PBS) pH 7.4 was prepared by mixing 0.1 M disodium hydrogen phosphate dibasic (Na_2_HPO_4_·2H_2_O) and sodium dihydrogen phosphate monobasic (NaH_2_PO_4_·H_2_O). This was used as the supporting electrolyte for all experiments, unless stated otherwise. The stock solution of CYP3A4 enzyme (87.26 µM), was prepared with PBS, pH 7.4 and stored at −80 °C until required. The enzyme solution was further diluted to the desired concentration with the same buffer solution when needed. All aqueous solutions were prepared with ultrapure water (18 MΩ/cm) obtained from a Purite water purification system (Veolia Water Purification Systems Ltd., Oxon, UK). All solutions used in the electrochemical studies were degassed with analytical grade argon.

### 2.2. Material Characterization Instrumentation

X-ray diffraction (XRD) was used to analyse the crystal and phase structure of the electrode materials via an X-ray diffractometer with Cu K radiation (=1.54060 Å) in 2∅ ranging from 20 to 80. The optical and structural properties of the synthesized material were investigated using the following techniques and their corresponding machines: Ultraviolet-Visible (UV-vis) Spectroscopy (Nicolett Evolution 100 (Thermo Electron Corporation, Johannesburg, South Africa)), Fourier Transform Infrared (FTIR) Spectroscopy (Perkin Elmer Spectrum 100 series Attenuated Total Reflection (ATR) FTIR spectrometer (Perkin-Elmer, Boston, MA, USA)), High Resolution Scanning Electron Microscopy (HR-SEM) (ZEISS ULTRA Scanning Electron Microscope fitted with an Energy Dispersive X-ray Spectrometer (EDS) (Carl Zeiss Microscopy GmbH, Jena, Germany)), and High Resolution Transmission Electron Microscopy (HR-TEM) (Field Electron and Ion Company (FEI) Tecnai G2 F2O X-Twin MAT 200 kV Field Emission Transmission Electron Microscope (FEI, Eindhoven, The Netherlands)).

### 2.3. Electrochemical Measurements

Cyclic voltammetry (CV) and differential pulse voltammetry (DPV) experiments were performed with a Palm-Sensor PT Trace 4.4 (PalmSens BV, Houten, The Netherlands) screen-printed electrode connector that was equipped with a Windows 10 integrated computer. The screen-printed carbon electrodes (ref. DRP-C110) were obtained from Metrohm-Dropsens (Asturias, Spain). CV was used to detect the various concentrations of LAPA (0–100 µM). The parameters for the experiment included a potential window of −1–1 V, at specific applied scan rates of 50 and 100 mV/s and 0.005 V potential step while detecting different concentrations of the drug. DPV curves were obtained within the voltage range of −0.6 to +0.6 V, at an applied scan rate of 50 mV/s. All experiments were performed in 0.1 M PBS or 10 mM DPBS pH 7.4 aqueous electrolytes, at room temperature, under aerobic and anerobic conditions. The bubbling of the electrolyte solution with N_2_ gas for 5 min and maintaining a N_2_ blanket over the solution allowed for anerobic experiments. Experiments for the optimisation and characterisation of the biosensor materials were performed by DPV and CV, respectively, in 10 mM DPBS buffer (pH 7.4) under anaerobic conditions. Biosensor preparation steps and biosensor responses to LAPA were studied by CV and DPV, respectively, in 10 mM DPBS buffer (pH 7.4).

### 2.4. Green Synthesis of Indium Nanoparticles

Indium nanoparticles (InNPs) were synthesized using a green synthesis method via ground coffee extract. The coffee beans (10 g) were finely ground and stirred for 1 h in a deionized water solution (100 mL) at 60 °C. The extract was then filtered and stored in a cool environment until it was used as a stabilizer and as a reducing agent for the synthesis of InNPs.

Indium (III) nitrate hydrate (3 g) was directly added to 30 mL of the extract, and the resulting mixture was heated and stirred at 80 °C for 2 h and, thereafter, cooled to room temperature. Once cooled, the reaction mixture was stored in the freezer till the sample was frozen. Thereafter, the sample was inserted in the freeze-dryer for 24 h. The brown formed precipitate was then incinerated in a muffle furnace at 800 °C for several hours till a yellow coloured powder was obtained, which confirmed the formation of the InNPs.

### 2.5. Synthesis of Poly(anilino-co-4-aminobenzoic Acid-Indium Nanoparticles) (Poly(ANI-co-4-ABA-InNPs)) Nanocomposites

The electrosynthesis of poly(ANI-co-4-ABA) was performed in a 0.5 M HCl solution at optimal experimental conditions in a conventional three-electrode cell. The co-polymer was formed through the electro-polymerization of 0.1 M (ANI + 4-ABA) and 0.5 M HCl [38]. The ratio for ANI:4-ABA was 1:2 in terms of molarity (i.e. 0.033:0.066 M). The poly(ANI-co-4-ABA-InNPs) nanocomposite was then formed by introducing 0.2% (5.5 × 10^13^ particles/mL) of the InNPs into the solution. The electro-polymerization of both the co-polymer and nanocomposite was performed by repeated cycling between the potentials −1 and 1.5 V and a scan rate of 50 mV/s for 7 cycles.

### 2.6. Preparation of SPCE||Poly(ANI-co-4-ABA-InNPs)|CYP3A4|PEG Nanobiosensor

**Scheme 1:**
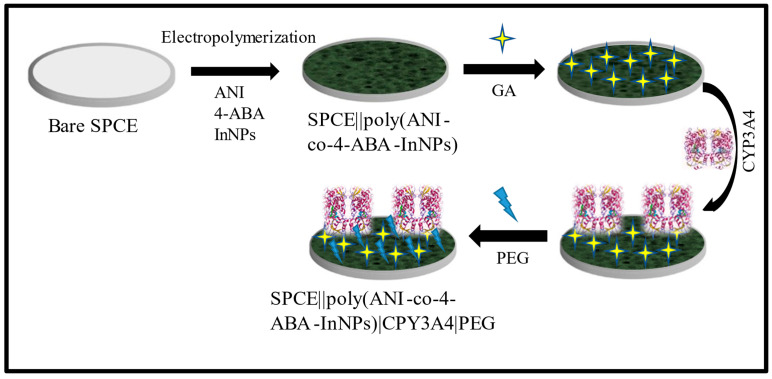
Schematic representation of the preparation of SPCE||poly(ANI-co-4-ABA-InNPs)|CYP3A4|PEG nanobiosensor.

The lapatinib electrochemical nanobiosensor system was prepared by first modifying the bare electrode according to the aforementioned method to form the resultant electrode SPCE||poly(ANI-co-4-ABA-InNPs), thereafter it was washed with 0.1 M PBS (pH 7.4) to remove any unbound material. Once the modified electrode dried, 20 µL of GA (0.1%) was added, which served as a cross-linker. After that, 20 µL of 100 µM CYP3A4 enzyme was drop coated and allowed to electrostatically attach to the modified electrode, the resultant electrode was sealed, dried, and stored in the refrigerator at 4 °C overnight, in a water-saturated atmosphere. Following that, polyethylene glycol (PEG) solution (5 mg/mL) was added as a blocking buffer to ensure non-specific binding for 1 h in a water-saturated atmosphere. During each step the modified electrode was carefully rinsed with water and PBS to remove any physically unbound material. The resulting SPCE||poly(ANI-co-4-ABA-InNPs)|CYP3A4|PEG modified electrode was stored at 4 °C in 0.1 M PBS (pH 7.4) when not in use. Scheme 1 represents the experimental procedure for the preparation of the nanobiosensor.

## 3. Results and Discussions

The first step in the green synthesis of the nanoparticles was to obtain a product consisting of two colours, initially brown and, then, yellow, after heating. These changes were reflective of the desired products being formed due to the completion of the chemical reactions. Additionally, the reaction conditions were optimized and the green synthesis of the InNPs was performed at different reaction times and at varying heating temperatures. By employing spectroscopic analyses, morphological studies, and electrochemical studies, a clear validation of the resultant data was achieved. It was concluded that the best product was achieved after 2 h of reaction time and at a temperature of 800 °C, which will be validated later in the article. The synthesized InNPs were combined with aniline and 4-aminobenzoic acid (1:2) to form an electropolymerized nanocomposite film which was used as a mediating platform to enhance the detection of LAPA.

### 3.1. Optical and Structural Properties of Poly(ANI-co-4-ABA-InNPs) Nanocomposite

#### FTIR and UV-VIS

FTIR spectroscopy has been used to analyze the as-synthesized materials’ vibration properties. The absorption bands and their positions are dependent on the crystal structure, the chemical composition, and the morphology of the material. The light-conducting technique, UV-vis spectroscopy, was also utilized to study the as-synthesized materials’ optical properties. InNPs that are green-synthesized are insoluble in water and can be partially soluble in ethanol. This makes them ideal for purification [39].

**Figure 1:**
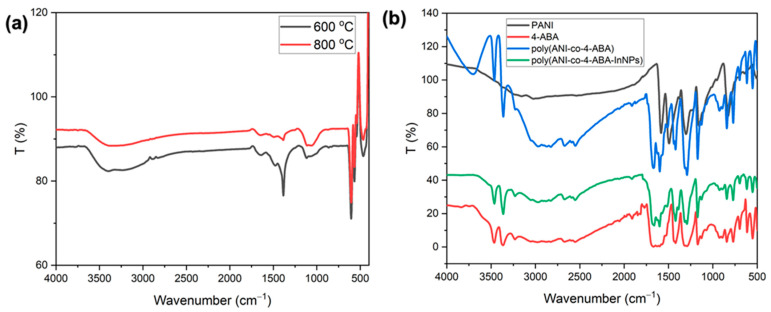
Typical FTIR spectra for (**a**) green-synthesized InNPs at a temperature of 600 °C and 800 °C and (**b**) the components, co-polymer and the co-polymer nanocomposite.

**Figure 2:**
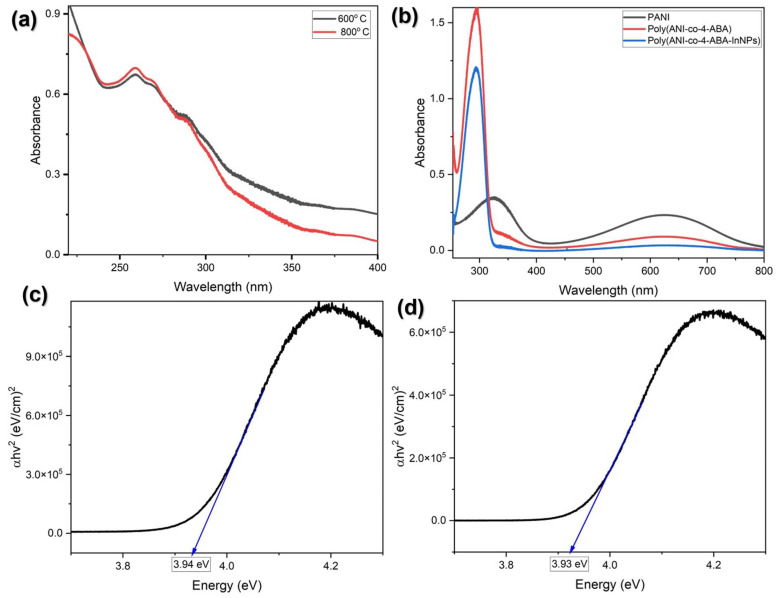
UV-vis absorption spectra of (**a**) green-synthesized InNPs at a temperature of 600 °C and 800 °C and (**b**) PANI alone, co-polymer, and the co-polymer nanocomposite. Tauc plot of (**c**) co-polymer (poly(ANI-co-4-ABA)) and (**d**) co-polymer nanocomposite (poly(ANI-co-4-ABA-InNPs)).

The FTIR and UV-vis spectra labelled as Figure 1a and Figure 2a showed the obtained spectral data of the as-prepared InNPs during optimization studies and, likewise, provided data revealing that InNPs were formed at both 600 °C and 800 °C. In the IR spectra, the presence of indium was observed based on the sharp vibrational bands that were observed in the 600–400 cm^−1^ range. The results of the IR spectra (Figure 1a) indicated that the temperature increase resulted in better results. In addition, the product’s purity was reflected in the spectra due to the presence of a primary InNP band and less scattering, which reflected on the purity of the product. The UV-vis data (Figure 2a) were also utilized to confirm the above statement, whereby the increase in temperature led to more clearly defined peaks and the spectra provided information about the prepared material before the process of forming the co-polymer nanocomposite [40].

Figure 1b represents the FTIR spectra of the starting materials and co-polymer nanocomposite. For 4-ABA (red line) the band around 3240–3500 cm^−1^ is attributed to characteristic N–H stretching vibration and the ammonium salts from the 4-ABA are ascribed to the small absorption bands between 2500 and 2700 cm^−1^, while the carbonyl group of the carboxylic acid found in 4-ABA is related to the band at 1668 cm^−1^. The deformation of different N–H bonds (4-ABA) correspond to bands that are found in the range of ~1500–1700 cm^−1^. Thus, the existence of C=N bonds are suggested due to the absorption bands above ~1610 cm^−1^, while the secondary amines (4-ABA) typically show weak absorption between 1100–1500 cm^−1^. The band that appears at approximately 1200 cm^−1^ is related to the single-band stretching of secondary aromatic amines, C–N. It supports the proposed mechanism for electro-grafting of certain materials. The existence of the absorption bands at 1700.26 cm^−1^ (reduced form) and 1770.26 cm^−1^ (oxidized form) highlights the carboxylic group being retained in the poly(4-ABA) chemical structure, thus, it was protected as it is an essential component for conjugation [41]. When considering PANI alone (black line) the most important absorption bands are 1634.62 cm^−1^ (C=O stretching), 1580.65–1572.33 cm^−1^ (C=C stretching), 1583.77–1200 cm^−1^ (C=C stretching in the benzenoid ring), 1493.23 cm^−1^ (C–C stretching in the aromatic ring), and 882.48–700 cm^−1^ (C–H out of plane bending modes), which indicates that an emeraldine structure is present [42]. Then, with the formation of the co-polymer and co-polymer nanocomposite, there are distinctive bands that appeared and shifted. The FTIR spectra of the co-polymer (poly(ANI-co-4-ABA)) and co-polymer nanocomposite (poly(ANI-co-4-ABA-InNPs)) show an absorption band at 842.22 and 841.5 cm^−1^, respectively, whereby the formation of the band can be attributed to the bending modes (C–H) of the aromatic rings in PANI. This finding supports the idea that aniline is present in both the co-polymer and co-polymer nanocomposite [43]. Additionally, the band assigned to C=O can still be found in the co-polymer at 1643.94 cm^−1^, confirming the successful introduction of 4-ABA and, then, with a slight blueshift (1642.48 cm^−1^) seen by the green curve as compared to that in the blue curve, which is the co-polymer alone, indicating the incorporation of the nanocomposite. The bands at 1642–1662 cm^−1^ are mainly attributed to the C=O bond of the co-polymer, which is less intense than that of the homopolymer. Additionally, the band at 3463.11 cm^−1^ of both the co-polymer and co-polymer nanocomposite is sharper than that of PANI at the same position due to the merger of the two individual stretching vibrations from –OH and –NH_2_ in 4-ABA. The two small out-of-plane bending vibrations at 770.69 and 715.09 cm^−1^ for the co-polymer and 771.91 and 717.16 cm^−1^ for the co-polymer nanocomposite are attributed to the C–H bonds of the benzene rings. The formation of quinoid structures (blue and green line) from the benzenoid rings (black line) is confirmed by the disappearance of the band at 1500 cm^−1^ which corresponds to the C=C stretching [44]. The In–O stretching vibration of the InNPs is observed at the absorption bands at around 466.51 cm^−1^ [45]. The band frequency of a nanocomposite might be attributed to the interaction between the carboxylic groups of a co-polymer and the InNPs hydroxyl groups [46].

The FTIR absorption spectra of the co-polymer and co-polymer nanocomposite is analogous to that of PANI to some extent, implying that the aniline unit is predominant in the co-polymer over the additional materials. This presumption can be verified by comparing the relative intensity of the two bands at 794.46 and 715.09 cm^−1^. The former is attributed to the 1,4-disubstituted benzene rings in the formation of PANI and it is much stronger than the latter attributed to the 1,2,4-trisubstituted benzene rings in the co-polymer and co-polymer nanocomposite polymerization [47]. Additionally, since the electro-polymerization of these materials was performed in 0.5 M HCl, Cl^−^ ions are also incorporated into the co-polymer and co-polymer nanocomposite to neutralize the positive charge delocalized over the co-polymer chains [48]. Thus, Cl^−^ ions are incorporated into both the co-polymer and co-polymer nanocomposite, and the corresponding vibrations of Cl^−^ are present between 850–550 cm^−1^. The FTIR spectra of the mixtures show that the films exhibited some absorption in the region around the 0.0 V reduction. This is feasible since the films were collected at this level. However, the absorption bands produced by carboxylic acid groups (1700 cm^−1^) are not visible in the spectra. It is possible that the presence of substituted monomer in the solution is small. However, the spectra shows that the presence of the PANI in the solution changed the film’s growth characteristics [49].

The UV-vis spectra in Figure 2a have a prominent absorbance peak in the range of 250–300 nm, which indicates the material’s good absorbance. Furthermore, an increase in the torsion angle between phenyl rings that are close to one another with respect to PANI affects the carboxylic acid groups [50], resulting in a lower extension of the conjugation of all the materials shown in the figure. Two absorption bands are observed for PANI alone, which represents the benzenoid (π–π* transition) and quinoid rings [51] at 330 nm and 635 nm, respectively. Along the polymer backbone, its conjugation is related to the π–π* transitions [52]. Then, for the co-polymer and co-polymer nanocomposite there are three distinct absorption bands (red and blue lines, respectively). In the polymeric chain the extended conjugation between adjacent rings results in the band at about 300 nm (π–π* transition). Then, due to the—COO^−^ group having non-bonding electrons [53] we observe a shoulder peak at about 350 nm (*n*–π* transition). The third wavelength band at ~650 nm corresponds to the π polaron transition. In Figure 2b the UV–vis spectra of PANI, poly(ANI-co-4-ABA), and poly(ANI-co-4-ABA-InNPs) are similar. However, when considering the co-polymer graph there are some slight differences that occur. The signals that are specific to a particular area appear at a lower wavelength than those in PANI. This is due to the co-polymers being less conjugated [54] when compared to PANI. The *λ*_max_ of this wavelength band depends on the co-polymer oxidation and the carboxylate group’s steric hindrance can affect the co-planarity of the π system. According to data from UV-vis, the incorporation of InNPs units in the co-polymer decreases the absorbance [55], which is in line with the FTIR observations. Additionally, the positions of the bands are blue shifted on addition of InNPs in the co-polymer [56], indicating its effect on the band gap and likewise its polaron density. This shift indicates a redistribution of polaron density in the band gap of the co-polymer which is due to the impact of InNPs [57]. The optical conductivity of the co-polymer increases in the presence of InNPs, due to the decrease in the optical band gap [58]. Therefore, the incorporation of InNPs to a co-polymer host causes the localized states to increase, which directly affects its optical band gap [59].

Furthermore, Figure 2c,d displays the Tauc plots for determining the band gaps of the co-polymer and the co-polymer nanocomposite. It is evident that the incorporation of the InNPs decreased the band gap, which is due to its small particle size and, thus, it will have a lower number of atoms/molecules, which leads to narrower conduction and valence bands due to the lower overlapping of orbitals of atoms. The results indicate that the formation of a nanocomposite can reduce the gap between the components electrochemical bands, likewise, the band gap confirms the formation of the nanocomposite [60]. This makes it an ideal material for biosensing applications.

### 3.2. Morphological Properties of Poly(ANI-co-4-ABA-InNPs) Nanocomposite

#### 3.2.1. TEM and XRD

**Figure 3:**
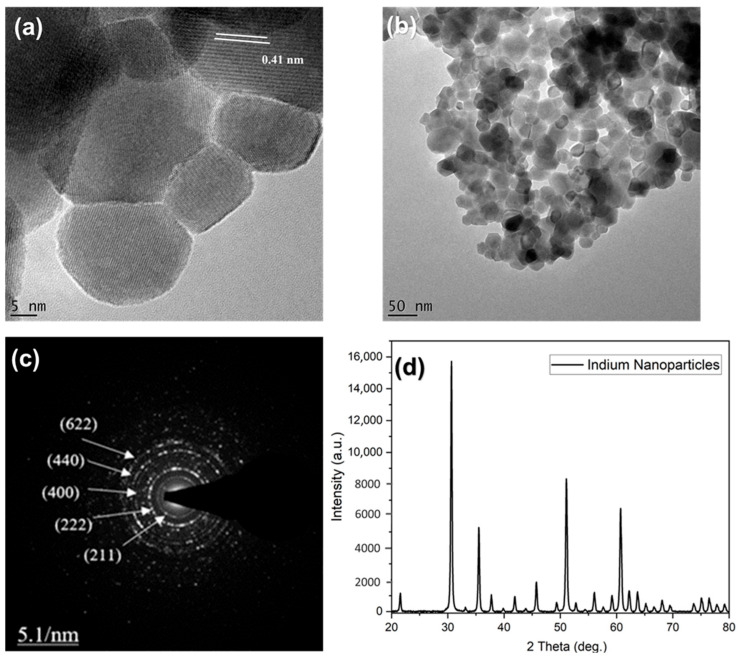
Transmission electron microscopy (TEM) images of green-synthesized InNPs at (**a**) 5 nm and (**b**) 50 nm magnification. The SEAD pattern for (**c**) green-synthesized InNPs, where the spherical dark zones correspond to InNPs in the bright amorphous matrix. XRD spectrum of (**d**) green-synthesized InNPs, recorded at room temperature. Debye–Scherrer relations and the 400-line width of XRD peak were used to the determine the average particle size (~27 nm).

A transmission electron microscope was used to study the various characteristics of the InNPs, such as its shape, size, morphology, and distribution. The images in Figure 3a,b show the characterization of the prepared InNPs. In Figure 3d, the XRD plot of the InNPs crystalline structure is shown, whereby the specimen was prepared by dispersing the powder onto a quartz specimen holder. The data collected during the phase analysis were subtracted from the background [61]. This procedure was used to determine the optimal phase for conducting a crystallographic study of the InNPs with zero background [62].

In Figure 3a, the dark and light lines that appear on one of the crystal planes are called lattice fringes. These periodic fringes are formed by diffracted waves that are transmitted from the atomic lattice planes of the crystalline material. The measurements of InNPs revealed a lattice spacing of 0.41 nm, which is attributed to the crystal plane of indium (211) [63]. The TEM images display polydisperse nanoparticles (polycrystalline), with some of them showing uniform size and shape; the possible reason for this is that during the synthesis process there might not have been an even distribution of heat on the nanoparticles, hence, the different sizes and shapes are seen [64]. The TEM image in Figure 3b shows the formation of dark spots of agglomerated crystallites. The corresponding selected area electron diffraction (SAED) patterns (Figure 3c) of the nanocrystalline indium sample represent the crystallinity of the material. Thus, this pattern features diffraction rings that are characteristic to the high crystallinity of the as-prepared InNPs. The SAED of the nanoparticles is consistent with the structure of indium, as it features ring patterns assigned to the specific planes that correspond with the XRD pattern (Figure 3d), where the brighter diffraction points (222) and (440) in the SAED pattern also correspond to the main peaks in XRD. Based on the images, one can deduce that the crystallites have a random distribution in their size and shape. However, there are short distances between individual nanoparticles [65], which indicates that the particles were not well dispersed and were found to be agglomerated, due to weak forces adhering the particles to each other [66]. The uniformity of the nanoparticles is good, and the nanoparticles formed were predominately globular and polydisperse. The nanoparticle sizes range between 20–50 nm, where many of them average in the 20–30 nm particle size range.

From Figure 3d, the XRD pattern exhibits some prominent peaks. These sharp peaks indicate that the nanoparticles have good crystallinity. The phase purity of the as-prepared InNPs is good and all diffraction peaks are perfectly indexed to the indium metal and globular phase structure with lattice parameters of a ¼ = 10.12 Å and Z ¼ = 16. In the obtained results, the following information was derived: the diffracted peaks acquired at diffraction angles 2θ of 21.40, 30.80, 32.96, 35.5, 38.40, 39.17, 41.30, 44.50, 51.0, 52.0, 56.58, 57.0, 62.0, 65.90, 74.90, and 79.20 matched to (211), (222), (101), (400), (411), (110), (332), (431), (440), (420), (200), (422), (622), (444), (800), and (653), respectively.

Based on the information provided from the XRD and using Debye–Scherrer relations Equation (1), one can determine the crystalline sizes and, thus, the average sizes.

(1) D=0.9λ/βcos∅

In the equation, D is the crystal size, 0.9 is the shape factor, λ is the wavelength of X-ray, β is the full width at half maximum of the peak in radians, and ∅ is the Bragg’s angle in radians.

The average size of the InNPs was determined using computational software (Microsoft Excel 15.0) and the above data. Thus, the average crystallite size was determined to be 27.311 nm. The average size obtained by this formula is in good accordance with those obtained from SEM and TEM images as well as literature as it has been reported that the size of InNPs is usually within the range of 6–40 nm [67,68].

#### 3.2.2. SEM and Energy-Dispersive X-ray (EDX)

**Figure 4:**
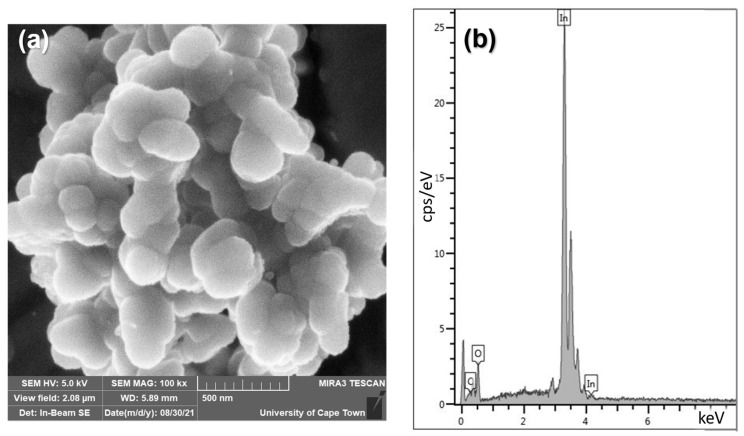
Scanning electron microscope (SEM) image (**a**), and SEM-based energy-dispersive X-ray (EDX) spectrum (**b**)**,** of green-synthesized InNPs in the time of 2 h.

Scanning electron microscopy (SEM) was used to study the surface topography and morphology of InNPs. The SEM images (Figure S1 in Supporting Information) showed the obtained morphology of the as-prepared InNPs during optimization studies. Additionally, it was determined that with an increase in synthesis time, there was a more prominent formation of the nanoparticles as well as a gradual increase in the number of nanoparticles. As such, it was observed that the increase in the reaction time led to the particles being more nanosized [69]. Furthermore, the energy-dispersive X-ray spectrum (EDX) of InNPs is represented in Figure 4b. This is used to analyze their chemical composition.

The synthesized InNPs shown in Figure 4a were well defined and had sizes similar to one another. The images revealed that the nanoparticles had a spherical shape and were composed of aggregates. Furthermore, the SEM images showed that the particles were often agglomerated. Due to the agglomeration of the grains, the larger particles were able to form [70]. The sizes of the synthesized InNPs were estimated to be around 20–40 nm, which strongly agrees with the TEM data. The edges and corners of the particles were also visible in the image. In addition, the InNPs featured a rugged surface, which has been previously reported [71].

Indium metal peaks can be seen in Figure 4b, which supports the presence of this element in the sample [72]. In the figure, we see that the only other elements present in the sample were carbon (C) and oxygen (O). However, the carbon element was from the carbon strip used for SEM analysis and the oxygen (O) element can be traced back to the reactor chamber’s residual oxygen [73]. This implies that the process of reducing the indium salt precursor’s oxide-reduction has been completed. The lack of presence of any other elements also supports the significant chemical purity [74] of the InNPs.

**Figure 5:**
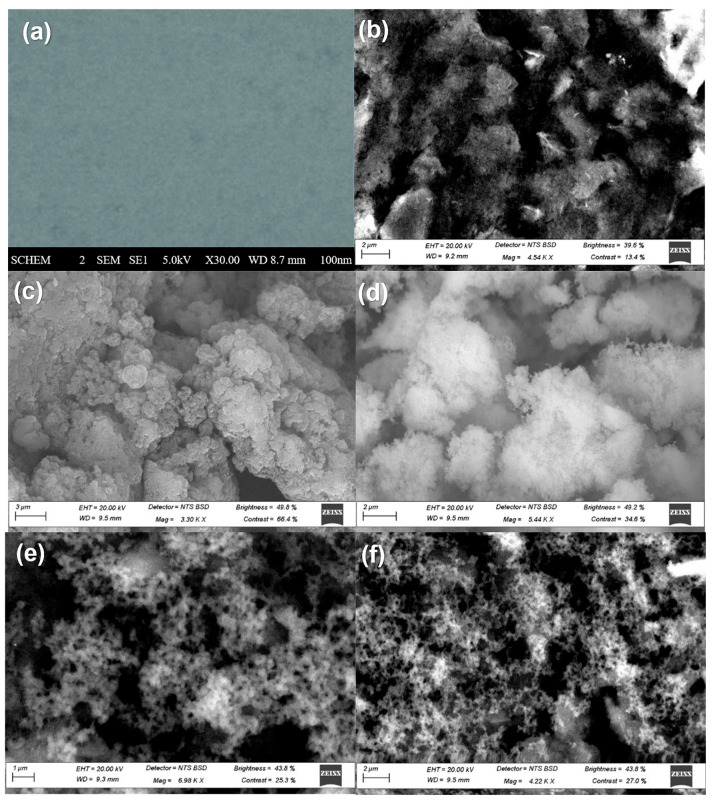
SEM images of the electrode platforms on SPCEs: (**a**) bare SPCE, (**b**) electro-grafted 4-ABA, (**c**) electropolymerized PANI, (**d**) electrodeposited indium nanoparticles, (**e**) electropolymerized poly(ANI-co-4-ABA), and (**f**) electropolymerized poly(ANI-co-4-ABA-InNPs).

Figure 5a–f contain the SEM images of the (a) unmodified SPCE, (b) electro-grafted 4-ABA, (c) electropolymerized PANI, (d) electrodeposited indium nanoparticles, (e) electropolymerized poly(ANI-co-4-ABA), and (f) electropolymerized poly(ANI-co-4-ABA-InNPs) on SPCEs. The electro-polymerization process led to a deposition of a material evenly distributed on the electrode surface. These images show the various types of spherical particles that were compressed [75]. Most of them formed globular compact structures that indicated the crystalline structure of the sample. Additionally, the grains of the nanoparticles were found to be interconnected with each other, which indicates that they have enough binding energy to combine with neighboring grains or molecules [54]. Pure PANI obtained via the chemical oxidation route is shown in Figure 5c, whereby we see that the particles have spherical agglomeration without uniform packing [76]. Thus, based on the results obtained, the high concentration of aniline resulted in the diameters of the PANI increasing and the molecule surface became coarser, thus, there was a great growth in PANI. In the end, the mixture consisted of large-sized irregular particles and agglomerated PANI with larger diameters [77]. This is also true based on the observed images illustrating the co-polymer (Figure 5e) and co-polymer nanocomposite (Figure 5f). Air bubbles are known to create holes on the surface of an electrode due to its composition [78], which are observed on the electrode surface. Other possible causes for the formation of these holes are the presence of a solvent that gradually evaporated in the surroundings [79,80]. The presence of the co-polymer on the electrode’s surface can be seen in Figure 5e due to its thick and rough texture [81]. This is because the pores are being covered by the poly(ANI-co-4 ABA). In addition, the figure shows that the poly(ANI-co-4 ABA) completely covers the electrode’s surface. Figure 5f shows the SEM image of poly(ANI-co-4-ABA-InNPs), where the fine flacks of InNPs in the co-polymer (poly(ANI-co-4-ABA)) matrix were observed and seen to form a cluster morphology. Due to the presence of InNPs, the crystallinity of composite materials can be enhanced and explains why we see some smooth solid blocks [72]. The SEM image (Figure 5f) shows the nanocomposite is found to have a highly agglomerated globular-like structure [54].

### 3.3. Electrochemical Evaluation of Poly(ANI-co-4-ABA-InNPs) Prepared as Thin Films onto SPCEs

#### 3.3.1. Electrochemical Characterisation of Nanosensor

**Figure 6:**
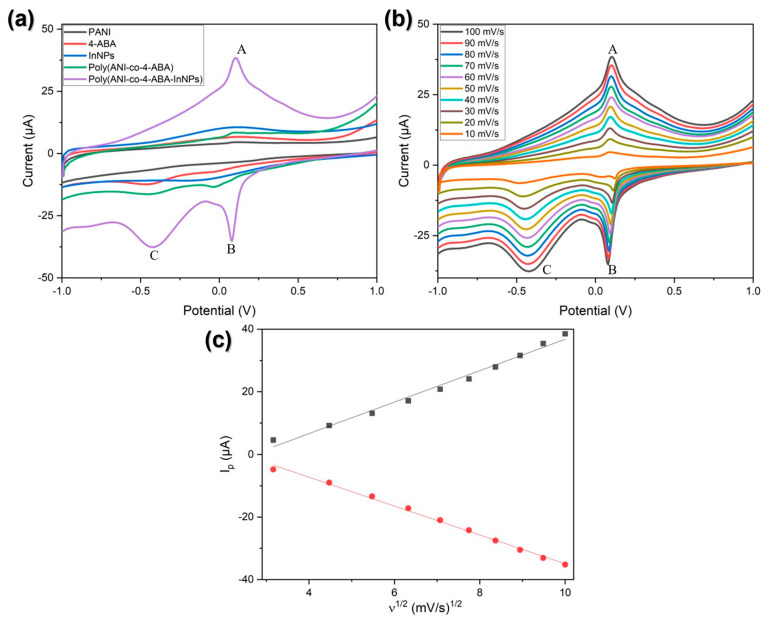
(**a**) CVs of electrode materials at 100 mV/s on SPCE. (**b**) The scan rate (ν) dependence CVs of poly(ANI-co-4-ABA-InNPs) modified electrode at 10 mV/s intervals (arrow refers to the CVs from inside outwards). 0.1 M PBS (pH 7.4) was used for the experiments under anaerobic conditions. (**c**) The Randles-Sevčik plots for the anodic Peak A (black line) and the cathodic Peak B (red line) of the CVs in (**b**).

The poly(ANI-co-4-ABA-InNPs) nanocomposite was prepared by electro-polymerization on SPCEs (Scheme S1 in Supporting Information). The electrodeposition was performed by CV to create a poly(ANI-co-4-ABA) co-polymer which was combined with green-synthesized InNPs to form the nanocomposite. The moiety of the co-polymer nanocomposite was then activated by an oxidation step to form a radical monomer [82,83]. Then, the reduction step occurred which was where the radical material was deposited onto the electrode after reversing the current [84]. This process was repeated many times to allow the formation of the polymer chain. The deposition was then observed with a rising current (Figure 6a). The potential window for deposition of the polymers at the working electrode was −1 to + 1 V at scan rate of 50 mV/s. This window has been reported as an ideal window to produce enough charge [85]. Likewise, the length of a polymer chain is affected by the rate at which a process is performed. If the rate is slow, the radicals deposited on the electrode will not be able to reach the desired destination. Moreover, the formation of a conductive nanocomposite was observed when the current intensity increased with more CV cycles [86]. The study on the nanocomposite’s electrochemical behaviour also made a significant contribution. Through this study, we were able to identify the various types of redox couples in the nanocomposite. It also provided useful information on the materials’ characteristics. The electrochemical response of the poly(ANI-co-4-ABA-InNPs) nanocomposite deposited onto SPCE (SPCE||poly(ANI-co-4-ABA-InNPs)) was presented using CV and DPV.

The CV profile of the co-polymer nanocomposite in Figure 6a exhibited the typical electrochemistry of the emeraldine salt [87], which supports the literature’s claim that the presence of 4-ABA did not alter the PANI electrochemistry [38]. Three pairs of redox peaks were observed for poly(ANI-co-4-ABA-InNPs) in 0.1 M PBS (pH 7.4). Based on the results obtained and previous studied literature, there is a shift in peak currents between PANI alone and Peak A (poly(ANI-co-4-ABA-InNPs)), which is due to two effects, that is, the carboxylic group’s withdrawal of electrons from the aromatic ring leads to the amine units becoming harder to oxidize. The other effect is that the steric effects of the ring’s other groups decrease the co-planarity of the amine units, making it harder to form rings. The reduction in conductivity makes it harder for the oxidation process to occur. This effect can also be seen in the positive shifts in the peak potentials of the co-polymer when 4-ABA units are being introduced [88]. Additionally, the larger peak currents of this redox couple of the co-polymer nanocomposite clearly demonstrate that it has an improved anion exchange ability [89] compared to PANI. The interconversion between quinone and hydroquinone or quinoneimine results in the second redox peak (B). The third redox peak (C) is linked to the interaction between pernigraniline and emeraldine. This event involves the exchange of protons between the solution and the co-polymer [48]. The anodic peak currents of the poly(ANI-co-4-ABA-InNPs) co-polymer nanocomposite was clearly observed, indicating high electroactivity [90]. The effect of InNPs on the poly(ANI-co-4-ABA) co-polymer’s oxidation process was also shown. They enhanced the platform’s electroactivity and caused a shift in the anodic peak’s potential from 0.1 V to 0.125 V (A) [47,91]. The main reason for the potential shift was due to the strong adsorption of the InNPs in poly(ANI-co-4-ABA). This resulted in the formation of more conjugated structures. Thus, the presence of the nanoparticles also promoted the transfer of electrons [88].

Furthermore, based on Figure 6a, the redox peak currents in the CVs of co-polymerization and the incorporation of InNPs are larger than those of the co-polymerization of poly(ANI-co-4-ABA) and the materials individually, indicating the growth rate of poly(ANI-co-4-ABA-InNPs) is faster than that of poly(ANI-co-4-ABA). This is attributed to the autocatalytic deposition of indium, since indium is introduced for the formation of the nanocomposite. In addition, the poly(ANI-co-4-ABA-InNPs) composite film showed peak currents that are much more defined. Thus, the increase in the current peak of a composite thin film was attributed to the presence of InNPs. The InNPs’ larger surface area allowed them to increase the loading of poly(ANI-4-co-ABA) on the electrode [92]. It also further validated their influence on the film’s oxidation process [93].

Figure 6b is the scan rate (ν) dependence cyclic voltammograms of poly(ANI-co-4-ABA-InNPs and Figure 6c is the Randles-Sevčik plots for Peaks A and B of Figure 6b. As can be observed in the CVs of Figure 6b, as the scan rate changes from 10 to 100 mV/s, there is a gradual increase in the peak currents. The peak potentials of Peaks A and C of Figure 6b did not change with changes in the scan rate, which confirms a surface-bound thin film of electroactive material. The peak potential of Peak B of the same figure shifts cathodically as the scan rate increases. This confirms the occurrence of charge diffusion within the polymeric chain of the co-polymer nanocomposite. In Figure 6c, it is noteworthy that the peak currents follow the Randles-Sevčik model, thereby indicating the occurrence of reversible electrochemistry of the diffusion-controlled process.

#### 3.3.2. Optimization Studies

**Figure 7:**
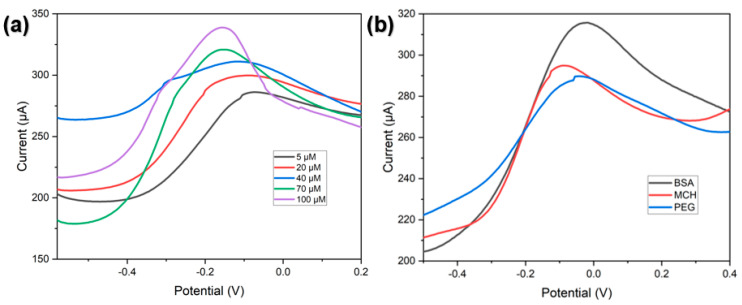
Differential pulse voltammograms (DPVs) for the optimization of: (**a**) CYP3A4 enzyme concentration and (**b**) blocking buffer (BSA, MCH and PEG) for SPCE||poly(ANI-co-4-ABA-InNPs)|CYP3A4|PEG nanobiosensor preparation. The measurements were performed in 10 mM DPBS buffer pH 7.4 under anaerobic conditions.

The graphs in Figure 7a,b illustrate the optimization parameters for the electrochemical detection of the drug LAPA, where the response currents were monitored as a function of enzyme concentration and type of blocking buffer.

Based on Figure 7a, we can see there was a fluctuation within the current response as the enzyme concentration increased. However, we see that the 100 µM produced the highest response. Thus, 100 µM was selected as the optimum enzyme concentration. The next optimization step included the monitoring of the current response towards different types of blocking buffers at their ideal concentrations. Then, based on Figure 7b, PEG (5 mg/mL) showed the lowest current response when compared to BSA (10 mg/mL) and MCH (1 mM). The goal of a blocking buffer is to reduce the non-specific signals generated by the binding of peptides or proteins. Thus, when analyzing the blocking buffer optimization results, we are looking at the blocking buffer that has the lowest current response, as this means that the specific blocking buffer blocked most of the signaling that was non-specific. Thus, the subsequent experiments were performed using PEG as the blocking buffer.

#### 3.3.3. Electrochemistry of the Step-by-Step Development of Nanobiosensor

A simple and sensitive method for LAPA detection was developed. For the first time, CYP3A4 was immobilized on SPCE||poly(ANI-co-4-ABA-InNPs) to form (SPCE||poly(ANI-co-4-ABA-InNPs)|CYP3A4) and used for the detection of LAPA by DPV. It is important to determine whether the immobilized enzyme is attached to the nanocomposite or not, and, also, to check if the resulting nanobiosensor has the necessary electroactive components for sensor application.

**Figure 8:**
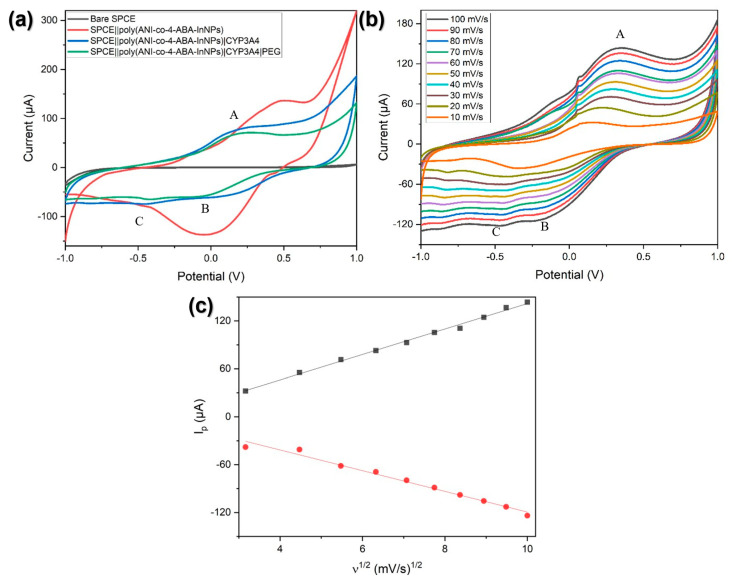
(**a**) CVs of the electrode systems at 50 mV/s scan rate, for the various stages of the nanobiosensor development. (**b**) Scan rate (ν) dependence CVs of SPCE||poly(ANI-co-4-ABA-InNPs)|CYP3A4|PEG nanobiosensor at 10 mV/s intervals (arrow refers to the CVs from inside outwards). (**c**) The Randles-Sevčik plots of the anodic Peak A (black line) and the cathodic Peak B (red line) of the CVs in (**b**). Conditions: 100 µM CYP3A4; 5 mg/mL PEG; and argon-degassed 10 mM DPBS buffer (pH 7.4). (For the voltammograms in (**a**,**b**), A represents the anodic peak, while B and C are the cathodic peaks of the redox processes.)

As seen in Figure 8a, the voltammogram of the bare SPCE (black line) does not have any redox peaks. The peak currents exhibited by the poly(ANI-co-4-ABA-InNPs)-modified electrode are a clear indication of its potential to improve the electrode’s conductivity and electron transfer rate [94]. Given the significant influence that the poly(ANI-co-4-ABA-InNPs) can have on the reaction’s kinetics, it is important that the environment is conducive to the CYP3A4 transfer. After incubation with the CYP3A4 enzyme, the current response exhibited by the modified electrode significantly decreased and shifted negatively. This indicates that the enzyme successfully self-assembled and formed on the modified electrode’s surface. The reduction in the current response exhibited by the modified electrode was caused by the transition from Fe^3+^/Fe^2+^ during the activity of the enzyme at the active site [95]. Thereafter, the blocking of the surface via the polyethylene glycol (PEG) molecule, which is bulky sized, also caused the current response to decrease [96]. This suggests that the nanobiosensor’s assembly is complete. One of the most crucial steps in the fabrication of nanobiosensors is the blocking of the unmodified sites. This process involves the use of PEG to cover the voids in the deficient enzyme’s overlay, which will increase the sensor’s charge transfer resistance and, thus, decrease the current [97]. Based on the results provided, it is evident that the anodic currents of SPCE||poly(ANI-co-4-ABA-InNPs)|CYP3A4|PEG were higher than those observed for the bare SPCE [98,99], where the stability and increased surface area of the nanocomposites are attributed to their properties. Furthermore, the presence of a co-polymer and CYP3A4 in its structure, which has carboxylic and amino acid active sites, can enhance the conductivity of the desired product [100].

The scan-rate dependence CVs of the SPCE||poly(ANI-co-4-ABA-InNPs)|CYP3A4|PEG nanobiosensor are depicted in Figure 8b. The peak potentials of Peaks A and B shift anodically and cathodically, respectively, as the scan rate increases. This is reminiscent of a diffusion-controlled reversible electron transfer process. Figure 8c indicates that the peak currents of Peaks A and B in Figure 8b follow the Randles-Sevčik paradigm, implying charge diffusion occurring along the composite polymer chain. As can be seen in Figure 8b, the peak potential of Peak C remains relatively invariable with scan rate, thereby confirming the presence of the surface-bound thin film electroactive platform. The peak separation between Peaks A and B (i.e., ΔE_p_ = E_p_B − E_p_A) for SPCE||poly(ANI-co-4-ABA-InNPs) (see Figure 6b) is 0.03 V, while that of SPCE||poly(ANI-co-4-ABA-InNPs)|CYP3A4|PEG is 0.54 V. The large ΔE_p_ for the nanobiosensor indicates (i) that the fast electron transfer reaction occurring at poly(ANI-co-4-ABA-InNPs) platform is coupled to the redox process of the CYP3A4 enzyme, and (ii) the presence of insulating species (PEG) in the bioelectrode.

### 3.4. Electrochemical Detection of Lapatinib Using the SPCE||Poly(ANI-co-4-ABA-InNPs)|CYP3A4|PEG Nanobiosensor

#### 3.4.1. Mechanism for the Electrochemical Detection of LAPA

**Scheme 2:**
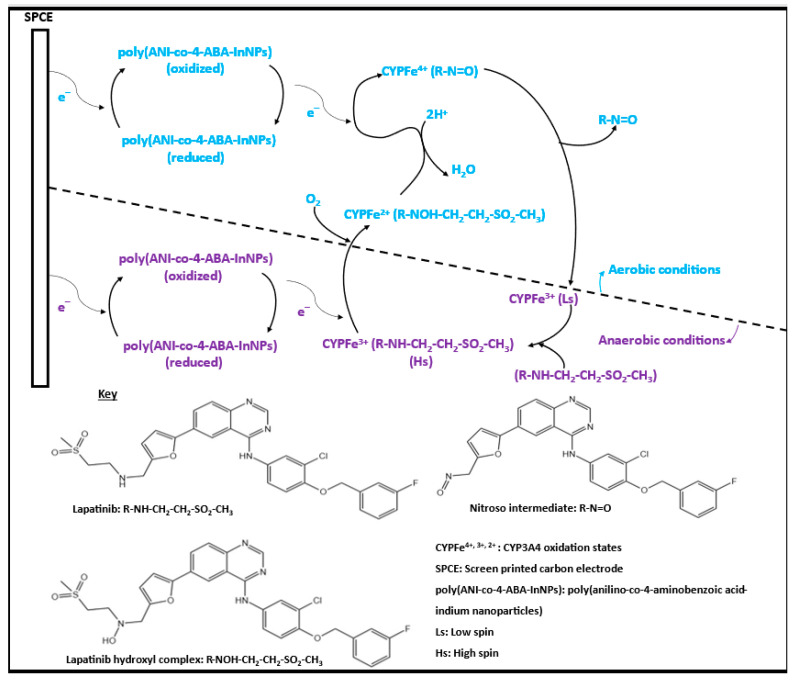
Schematics for the hydroxylation reaction of the SPCE||poly(ANI-co-4-ABA-InNPs)|CYP3A4|PEG LAPA biosensor. Broken line (**-----**) separates aerobic condition (blue colour) and anaerobic condition (purple colour) sections of the reaction scheme.

LAPA is a substrate-inhibitor of the CYP3A4 enzyme that is known to metabolize in the process of hydroxylation. This study reveals a new method for bioprocessing LAPA by combining the CYP3A4 enzyme with the prepared poly(ANI-co-4-ABA-InNPs) co-poly mer nanocomposite. The biosensing mechanism developed by this method is based on the reactions shown in Scheme 2 wherein the transformation of lapatinib to its oxidized form is carried out using the electrons and oxygen molecules [101]. Naturally, reduced nicotinamide adenine dinucleotide phosphate (NADPH) molecules provide electrons to the enzyme, however, in voltametric measurements, the current flow from the electrode provides these electrons [102,103,104]. Scheme 2 provides a single-electron reduction of the low-spin ferric enzyme Fe^3+^ to the high-spin ferrous enzyme Fe^2+^. The affinity of the enzyme for oxygen leads to the formation of a hydroxyl complex (CYP3A4 (Fe^2+^)). This process also triggers the release of a water molecule, which leads to the formation of a highly active nitroso intermediate (CYP3A4 (Fe^4+^)). In the case of the biotransformation of LAPA by the SPCE||poly(ANI-co-4-ABA-InNPs)|CYP3A4|PEG nanobiosensor, LAPA is oxidized to form a hydroxylamine which can be oxidized further to a nitroso intermediate, then, the intermediate can form a metabolic-intermediate complex (MIC) with the CPY3A4, which inhibits the activity of the enzyme. Huang et al. [105] described and showed the mechanism reaction for the formation of the nitroso intermediate from LAPA.

The use of the co-polymer nanocomposite for enhancing the transfer of electrons has been shown. Their electronic properties indicate that they can promote such reactions when utilized as an electrode material in certain electrochemical processes [106]. The detection mechanism is composed of a cyclic step that involves measuring the current produced by the enzyme after a substrate has been added to the sample. Thus, studies have shown that the retention of an enzyme or protein’s biocatalytic activity can enhance its electrochemical reactivity, which could render nanocomposites suitable for use in biological sensors [107]. The enhanced current effect of the co-polymer nanocomposite can be seen in Figure 8a, where the measured response of the bare electrode and the co-polymer nanocomposite electrode were compared. The cathodic peak in the figure depicts the coupling of a fast electron transfer reaction taking place at the electrode surface. Thus, the analytic performance of the nanobiosensor was improved.

#### 3.4.2. SPCE||Poly(ANI-co-4-ABA-InNPs)|CYP3A4|PEG Nanobiosensor for the Detection of LAPA

**Figure 9:**
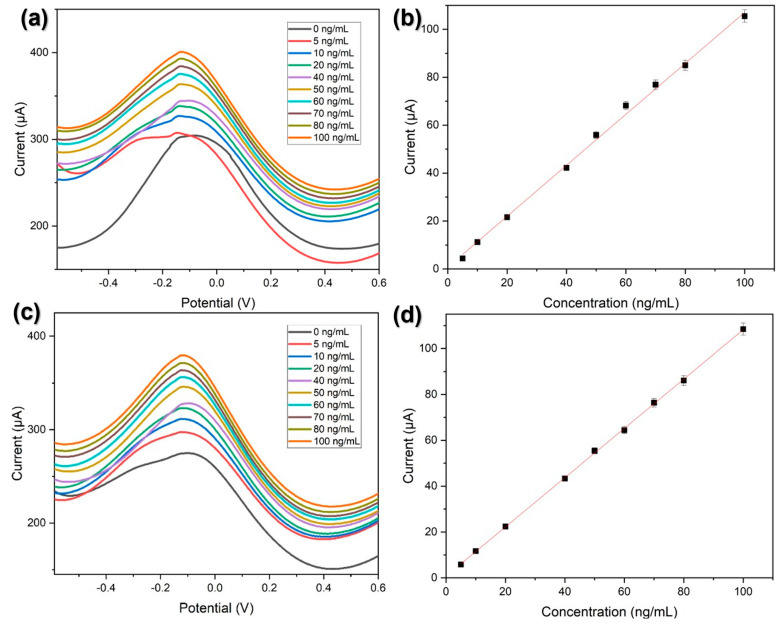
DPVs (at 50 mV/s) and the calibration plots for SPCE||poly(ANI-co-4-ABA-InNPs)|CYP3A4|PEG nanobiosensor responses to lapatinib in: (**a**,**b**) 10 mM DPBS buffer pH 7.4 and (**c**,**d**) human serum (diluted 10-fold in 10 mM DPBS buffer, pH 7.4). Each value in the calibration plots (i.e., (**b**,**d**)) corresponds to replicated experiments (n = 3) performed at −0.1 V under aerobic conditions, and the error bars indicate the RSD.

The electrochemical behavior of LAPA was studied using SPCE||poly(ANI-co-4-ABA-InNPs)|CYP3A4|PEG nanobiosensor and investigated using DPV. The respective responses of the SPCE||poly(ANI-co-4-ABA-InNPs)|CYP3A4|PEG nanobiosensor towards LAPA were investigated in 10 mM DPBS pH 7.4, and in human serum that was diluted 10-fold in 10 mM DPBS, under aerobic conditions at 50 mV/s. From Figure 9a,c, it is evident that the oxidation currents increased with increasing concentrations of LAPA in both physiological buffer solution and human serum. In addition, it is evident from the complementary calibration curves that there is a linear increase for different concentrations of LAPA, as shown in Figure 9b,d.

The linear calibration plots of the lapatinib nanobiosensor (SPCE||poly(ANI-co-4-ABA-InNPs)|CYP3A4|PEG) are shown in Figure 9b,d, in which a concentration range of 0–100 µM was studied. Two linear regions are defined in the calibration curve, and the differences in their slopes can be attributed to the varying LAPA concentrations. The number of sites on a sensor’s surface is higher in the low LAPA concentration [108]. This renders the sensor more sensitive to the molecules on its target. As the concentration of LAPA increases, the number of sites on the sensor’s surface decreases. This causes its sensitivity to decrease as it reaches a state of saturation [109]. The nanobiosensor amperometric responses to LAPA were fitted to the electrochemical Michaelis–Menten steady-state kinetics. Based on this, the apparent Michalis–Menten constant (K_M_^app^) and I_max_ were calculated to be 2.77 nM and 400.85 µA, respectively. The I_max_ value validates the electroactivity of the nanobiosensor and the low K_M_^app^ value confirmed that CYP3A4 was immobilized onto the electrode surface and that it maintained its catalytic properties and high enzymatic activity/affinity towards LAPA. Additionally, the calibration curves, sensitivity, limit of detection (LOD), and limit of quantification (LOQ) were determined from triplicate experiments. Sensitivity values of 0.654 and 1.0145 µA/(ng/mL) were determined for the nanobiosensor in DPBS and human serum samples, respectively. The sensor parameters for LAPA determination in human serum sample and buffer solution are presented in Table 1. The results of the study indicated that the procedure utilized for this drug was satisfactory and that it was within the desired drug ranges of 1 to 1000 ng/mL [110]. Thus, the overall analytical performance of the nanobiosensor shows it also holds promise for possible application in the detection of LAPA in pharmaceutical samples [111].

As listed in the table, the developed nanobiosensor exhibits a comparable, or even lower, LOD and LOQ values than most of the previously published works [120], although there are some detection methods that exhibit a lower LOD than our reported work. Nonetheless, these methods require large instruments, use complicated sensor fabrication processes, and cannot achieve rapid detection such as our findings in this study [121,122]. The obtained LOD and LOQ values of the nanobiosensor were lower than LAPA’s maximum steady state plasma concentration C_max_ (2.43 µg/mL), indicating that it can be used in real human samples. This also shows the nanobiosensors ability to be customized for higher concentrations, which makes it ideal for measuring LAPA. It also enables the detection of drugs in patients who are fast metabolizers, which means they can quickly remove their medication from their bodies. Thus, the device can be utilized for real-time monitoring [123].

#### 3.4.3. Control Studies of SPCE||Poly(ANI-co-4-ABA-InNPs) Responses to Lapatinib

**Figure 10:**
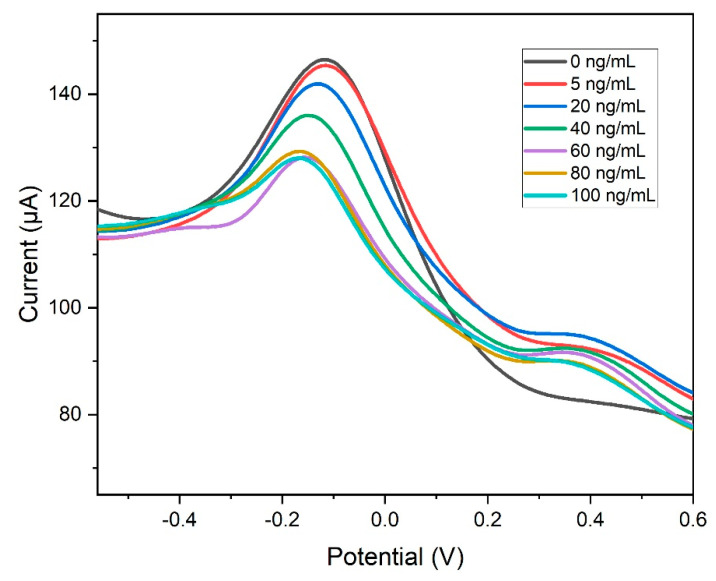
DPVs (at 50 mV/s) of SPCE||poly(ANI-co-4-ABA-InNPs) nanobiosensor responses to lapatinib in 10 mM DPBS buffer pH 7.4.

A control experiment was performed with poly(ANI-co-4-ABA-InNPs) nanoelectrode (i.e., it does not contain CYP4A4). The DPV plots of the responses of the nanosensor to LAPA are displayed in Figure 10. It can be seen that within the potential region of interest, the peak current of poly(ANI-co-4-ABA-InNPs) decreases as the concentration of LAPA increases. This implies that there is no direct reduction of LAPA at the electrode (within the response observation potential range). Rather, the adsorption of LAPA on the electrode surface form an insulating film that reduces the electroactivity of the poly(ANI-co-4-ABA-InNPs) nanoelectrode. This finding of the control experiment implies that the results reported in Figure 9 are due to the responses of SPCE||poly(ANI-co-4-ABA-InNPs)|CYP3A4|PEG nanobiosensor.

### 3.5. Selectivity, Stability, and Reproducibility of Lapatinib Biosensor

The stability of the bioelectrode, SPCE||poly(ANI-co-4-ABA-InNPs)|CYP3A4|PEG was evaluated. After a week of storage in 10 mM DPBS at an ambient temperature of 4 °C, the modified nanobiosensor maintained more than 80% of its original response, as shown in Figure S2 and Table S1 in Supporting Information. The results of the experiments indicated that the bioelectrodes exhibited a stable performance, as there was a less than 20% decrease in the current response. This also suggested that these devices could potentially be used for long-lasting storage.

**Figure 11:**
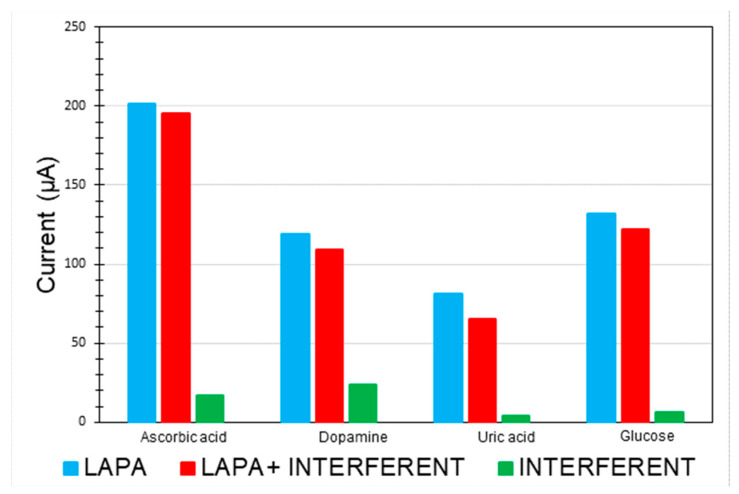
Selectivity of the SPCE||poly(ANI-co-4-ABA-InNPs)|CYP3A4|PEG nanobiosensor in the presence of 5 ng/mL LAPA and 50 ng/mL interferents: uric acid (UA), ascorbic acid (AA), glucose, and dopamine (DA) in DPBS (pH 7.4).

The sensitive nature of electrochemical nanobiosensors makes them ideal for detecting various analytes. Unfortunately, they can also be affected by other compounds. In the reduction of lapatinib, various compounds have been reported interfering with the analyte detection. Some of these include ascorbic acid (AA), glucose, uric acid (UA), and dopamine (DA). The possible interference effects of these substances on the electrochemical response of LAPA were studied on the prepared nanobiosensor (SPCE||poly(ANI-co-4-ABA-InNPs)|CYP3A4|PEG). The modified SPCEs were optimized in the aforementioned conditions and immersed in interference solutions (50 ng/mL) in triplicate analysis. From the interference studies (Figure 11), glucose has a smaller effect on the resulting nanobiosensor when compared to dopamine and ascorbic acid. This is due to a neutral charge which cannot be attracted by the polymer, which is a positive charge. In contrast to glucose, AA and DA will form anions in solution, so they are likely to be attracted by a polymer layer. Moreover, there have been various research efforts conducted showing that AA is one of the most severe interferents [124,125] and can interfere with methodologies involving redox reactions, likewise with DA since it coexists with AA [126]. Additionally, based on the structure of the interferents, there are functional groups present that could possibly contribute to the formation of amide bonds. Thus, it could have an impact on the current response of the analyte towards the modified electrode (nanobiosensor). Furthermore, in an enzymatic nanobiosensor, one of its main limitations is its signal reduction due to the presence of foul agents or interference from the sample’s chemicals. Moreover, CYP3A4 is a low-specificity CYP enzyme as it contributes to the metabolism of about 50% of market drugs [127]. Thus, due to the low specificity, we expect low selectivity. However, herein we show that the nanobiosensor response is also influenced by design parameters. Based on the data received, the modified enzymatic nanobiosensor still holds enormous promise. The oxidation peak current of LAPA slightly decreased in the presence of 10-fold interferents and had negligible interferences in the range of 0.8% to 1.09% (Table 2). This shows that the prepared electrochemical nanobiosensor can be used to determine LAPA. It also exhibited anti-interference capabilities.

## 4. Conclusions

Herein, a novel and simple nanobiosensor has been studied for the detection of a breast cancer drug, lapatinib. The nanobiosensor was developed by, firstly, synthesizing indium nanoparticles via a green synthetic route based on the use of a coffee extract as the reducing agent. We then electrosynthesized a co-polymer nanocomposite using aniline, 4-aminobenzoic acid, and the prepared nanoparticles. The characterization process was performed using various methods such as HR-TEM, HR-SEM, FTIR, and UV-vis. The properties of the materials were also investigated using electrochemistry to evaluate the sensor development and its performance. The study illustrated that the incorporation of the nanocomposite onto the platform suggested a stable attachment point for the cytochrome P3A4 enzyme. The platform also functioned as an efficient electron mediator between the modified electrode’s surface and the enzymes redox center. The results of the study revealed that the proposed nanobiosensor (SPCE||poly(ANI-co-4-ABA-InNPs)|CYP3A4|PEG) was able to successfully provide a catalytic response towards the detection of lapatinib. Furthermore, the detection limit of the sensor (13.212 ng/mL) falls within the LAPA detection range, thus highlighting that it can be used to trigger the drug’s conversion into active metabolites. Likewise, the LOD and LOQ (40.04 ng/mL) obtained for the developed nanobiosensor was found to be lower than the maximum plasma concentration (C_max_) of lapatinib (2.43 µg/mL). Thus, the device will be able to be used in real-time monitoring of the drug in patients. It could also be used in a clinical setting to detect the presence of drugs in high plasma concentrations. The nanobiosensor’s high sensitivity and ability to detect the lower concentrations of drugs makes it an ideal choice when monitoring low-dose medications for cancer treatment.

## Figures and Tables

**Table 1:** Comparisons with chromatographic LOD and LOQ values for the detection of lapatinib.

Detection Technique	LOD (ng/mL)	LOQ (ng/mL)	Reference
SPCE||poly(ANI-co-4-ABA-InNPs)|CYP3A4|PEG in DPBS	13.212	40.04	This work
SPCE||poly(ANI-co-4-ABA-InNPs)|CYP3A4|PEG in human serum	18.6	56.26	This work
Reverse-phase HPLC	265	884	[112]
HPLC-UV/vis	100	200	[113]
Validated LC (chemometric principles)	500	1000	[114]
Ultra-performance liquid chromatography (UPLC)	60	180	[115]
LC-MS -HPLC	10	40	[116]
RP-HPLC-DAD	1000	5000	[117]
LC-ESI-MS/MS	4.2	15	[118]
HPLC	125	300	[119]

**Table 2:** Interference effects on LAPA oxidation peak current at modified SPCE.

Interferent	LAPA (ng/mL)	Interferent (ng/mL)	LAPA Response (µA)	Interferent Response (µA)	LAPA + Interferent Response (µA)	Level of Interference (%)
Ascorbic acid	5	50	201	17	195	1.013
Dopamine	5	50	119	24	109	1.09
Uric acid	5	50	81	4	65	0.8
Glucose	5	50	132	6	122	1.08

## Data Availability

Publicly available datasets were analyzed in this study. This data can be found here: DSpace Home (uwc.ac.za).

## Supplementary Materials

The following supporting information can be downloaded at: https://www.mdpi.com/article/10.3390/bios13090897/s1.
