# Supplementary material for: Novel Cytochrome P450-3A4 Enzymatic Nanobiosensor for Lapatinib (a Breast Cancer Drug) Developed on a Poly(anilino-co-4-aminobenzoic Acid-Green-Synthesised Indium Nanoparticle) Platform
Source: Biosensors (Basel). 2023 Sep 21;13(9):897. doi: 10.3390/bios13090897 (PMC10527071; doi:10.3390/bios13090897)
Supplement: Supplementary file 1 [file biosensors-13-00897-s001.zip › biosensors-2544649-supplementary.pdf]

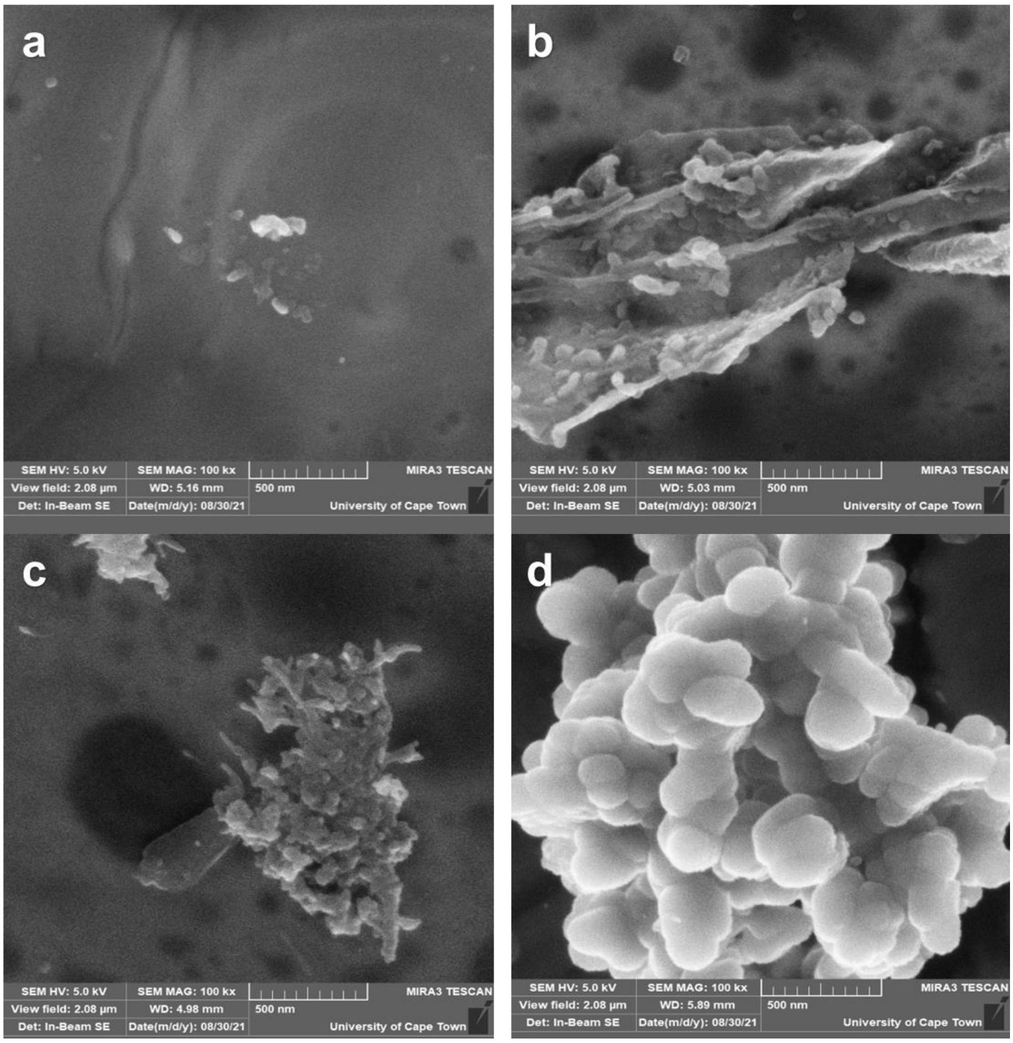

Figure S1. Scanning electron microscopy (SEM) analysis of green-synthesized indium nanoparticles in the time of: **(a)** 30 min, **(b)** 1 h, **(c)** 1.5 h and **(d)** 2 h.

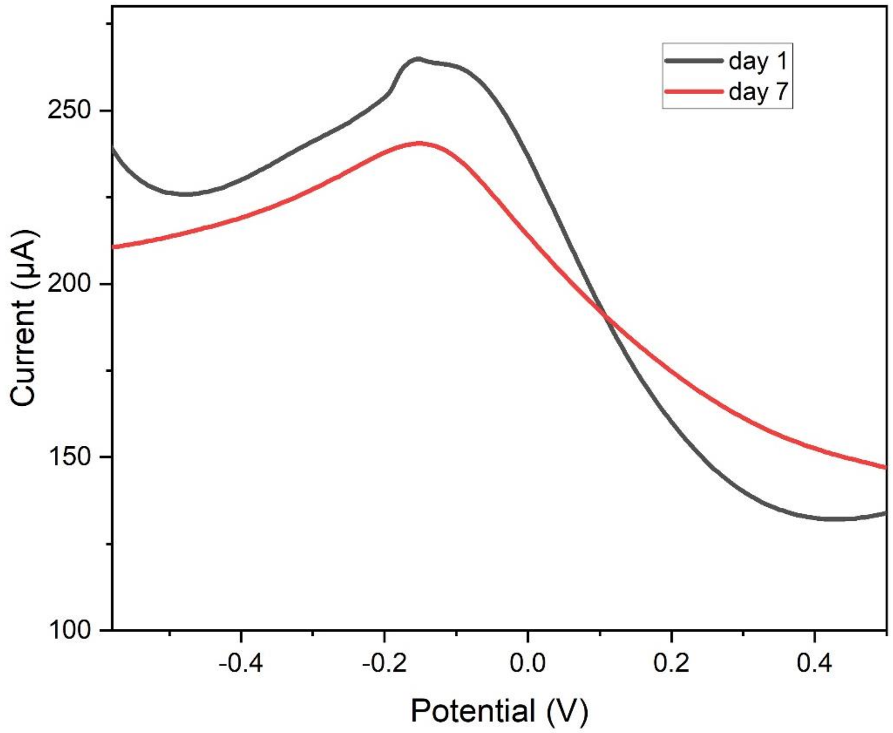

Figure S2. Stability studies: of the biosensor at day 1 and day 7 in DPBS at pH 7.2.

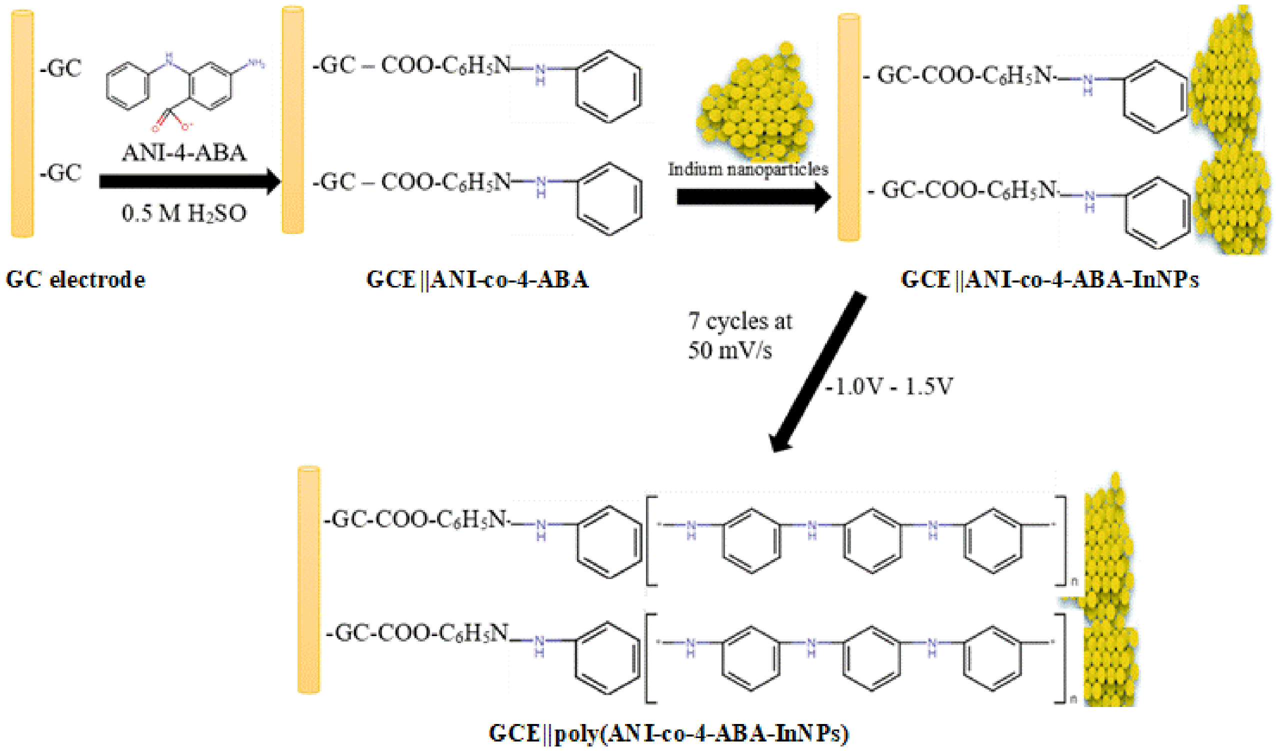

Scheme S1. Proposed schematic representation of ANI-4-ABA-InNPs polymerization on GC electrode to produce poly(ANI-co-4-ABA-InNPs).

Table S1, Depicting the stability studies of the SPCE||poly(ANI-co-4-ABA-InNPs)|CYP3A4 biosensor based on the peak current response.

	**Run 1 (μA)**	**Run 2 (μA)**	**Run 3 (μA)**	**Average**	**Retained current**
SPCE||poly(ANI-co-4-ABA-InNPs)|CYP3A4	323	341	400	355	80.4%
SPCE||poly(ANI-co-4-ABA-InNPs)|CYP3A4 stored at 4 ℃ in 10 mM phosphate buffer solution for one week	486.7	143.7	240.4	291.5	
